# Machine learning-based forecasting of air quality index under long-term environmental patterns: A comparative approach with XGBoost, LightGBM, and SVM

**DOI:** 10.1371/journal.pone.0334252

**Published:** 2025-10-08

**Authors:** Sevtap Tırınk

**Affiliations:** Department of Medical Services and Techniques, Environmental Health Program, Iğdır University, Iğdır, Türkiye; National Institute of Informatics, JAPAN

## Abstract

Air pollution is a global problem that threatens environmental sustainability and severely affects public health. Monitoring air quality and predicting future pollution levels are critical for creating effective environmental policies and enabling individuals to take precautions against air pollution. This study presents a long-term assessment of daily Air Quality Index (AQI) prediction using machine learning models based on meteorological and pollutant data collected in eastern Türkiye from 2016 to 2024. The dataset includes four major air pollutants (PM₁₀, SO₂, NO₂, O₃) and five meteorological variables (temperature, precipitation, relative humidity, wind direction, wind speed). Three models—eXtreme Gradient Boosting (XGBoost), Light Gradient Boosting Machine (LightGBM), and Support Vector Machine (SVM)—were evaluated using the coefficient of determination (R²), root mean square error (RMSE) and mean absolute error (MAE) as performance metrics. Among these, XGBoost achieved the highest prediction accuracy (R² = 0.999, RMSE = 0.234, MAE = 0.158). The results demonstrate that ensemble-based machine learning approaches, particularly XGBoost, can effectively model AQI fluctuations using environmental predictors. These results provide valuable insights for air quality forecasting systems and suggest practical implications for regional air pollution management and early warning systems, supporting public health protection and the development of environmental health policies.

## Introduction

Air is an indispensable resource for the sustainability of life, and its quality is critical to human health and the environment. However, air pollution has become a serious global environmental problem that negatively affects atmospheric quality and public health [1,2]. Rapid industrialization and urbanization have significantly increased the concentration of harmful substances in the atmosphere, especially in industrial areas, resulting in increased pollutant emissions [3,4]. This trend has increased public awareness and concern about air quality, as well as triggered calls for the development of effective air quality management and pollution control strategies [5].

The urbanization process plays a decisive role in air pollution levels. The spatial arrangement of urban areas affects the distribution and concentration of pollutants, causing denser urban environments to be exposed to higher pollution levels, usually due to traffic emissions and industrial activities [4,5]. In the Iğdır province of Türkiye, rapid economic expansion and urbanization processes also lead to a severe deterioration in air quality. In many regions of Türkiye, especially in the winter months, using fossil fuels for heating is one of the main factors increasing air pollution. In regions such as Iğdır, this situation becomes more pronounced in the winter months, and air quality deteriorates significantly [6,7].

The effects of air pollution on public health are profound. Various studies have shown that exposure to major air pollutants — including particulate matter (PM₂.₅ and PM₁₀), sulfur dioxide (SO₂), nitrogen oxides (NOx), carbon monoxide (CO), and ozone (O₃) — is associated with serious health problems such as respiratory diseases, cardiovascular diseases, and even cancer [8,9]. In this regard, Kumar et al. [10] highlighted how particulate matter, together with meteorological conditions, critically contributes to poor air quality and adverse health outcomes. The world health organization emphasizes the urgent need for comprehensive monitoring and management strategies because of premature deaths from air pollution [11]. Increasing urban populations makes air quality protection more critical, necessitating addressing both local and transboundary pollution sources [12]. In this context, authorities are trying to combat air pollution by developing long-term strategies. However, since these solutions require large-scale implementation, they can be costly in terms of time and financial resources. Providing air quality estimates and air quality index (AQI) values is important for individuals to develop protection strategies [13]. Air quality is influenced by the interactions among economic development, urban planning, and public health. Forecasting air pollution can support local governments and vulnerable groups (e.g., individuals with respiratory diseases, pregnant women, and children) by indicating when and where air quality may deteriorate and enabling timely protective measures.

As a widely used index, the AQI provides an overall assessment of environmental air quality. It expresses the overall air quality with a single numerical value by combining the concentrations of specific pollutants (Particulate Matter smaller than 2.5 micrometers (PM_2.5_), Particulate Matter smaller than 10 micrometers (PM_10_), Ozone (O₃), Carbon Monoxide (CO), Nitrogen Dioxide (NO₂), and Sulfur Dioxide (SO₂)) [14,15]. The Turkish National Air Quality Index (TNAQI) is the version of AQI developed by the US Environmental Protection Agency (EPA) and adapted to national legislation and limit values [16]. On a scale from 0 to 500, the AQI is divided into six categories – good, moderate, unhealthy for sensitive groups, unhealthy, very unhealthy, and hazardous – with corresponding health warnings provided in Table 1 [16]. As the AQI value increases, the threat to human health also increases.

**Table 1:** Value ranges of AQI and corresponding health warnings.

Values of index	Levels of concern	AQI colour	Description of air quality
0–50	Good	Green	Air quality is satisfactory and air pollution poses little or no risk.
51–100	Moderate	Yellow	Air quality is acceptable, but some pollutants may pose a moderate health concern for a small number of people who are unusually sensitive to air pollution.
101–150	Unhealthy for sensitive groups	Orange	Health impacts may occur for sensitive groups. The general public is unlikely to be affected.
151–200	Unhealthy	Red	Everyone may begin to experience health effects, with serious health effects for sensitive groups.
201–300	Very unhealthy	Purple	May constitute a health emergency. The entire population is likely to be affected.
301–500	Hazardous	Maroon	Health alert: Everyone may experience more serious health effects.

The relationship between air quality and meteorological factors is shaped by complex chemical and dynamic atmospheric reactions. Concentrations of air pollutants are highly sensitive to meteorological conditions such as wind speed and direction, relative humidity, and temperature, which have been shown in various studies to directly affect local air pollution levels [7–17]. Sekula et al. [18] evaluated the effect of atmospheric circulation on air quality, emphasizing the importance of humidity and temperature gradients, especially in the lower troposphere. Therefore, meteorological conditions should be taken into account in air quality assessments and forecasting, since they critically influence pollutant dispersion rather than being controllable factors for improving air quality.

In recent years, the use of machine learning algorithms in air quality prediction has become increasingly widespread, and significant progress has been made in this field. Studies conducted in this field in recent years are summarized in Table 2, where model performances are primarily assessed using Root Mean Squared Error (RMSE), Mean Absolute Error (MAE), and the coefficient of determination (R²). The table includes a variety of machine learning methods such as Random Forest (RF), Random Forest Regression (RFR), Cubist Regression (CR), Decision Tree (DT), Multilayer Perceptron (MP), Gradient Boosting (GB), Linear Regression (LR), Stacked Models (SM), k-Nearest Neighbor (KNN), Light Gradient Boosting Machine (LightGBM), Support Vector Regression (SVR), Long Short-Term Memory (LSTM), Logistic Regression (LogR), Artificial Neural Network (ANN), Improved Long Short-Term Memory (ILSTM), and Bayesian Regularized Neural Networks (BRNN). Meteorological variables are abbreviated as Temperature (T), Precipitation (P), Relative Humidity (RH), Wind Direction (WD), and Wind Speed (WS). This progress results from collaborative efforts, as various studies examine the methods developed to increase the effectiveness of machine learning techniques in air quality prediction [19–21].

**Table 2:** Summary of studies applying machine learning models to predict the AQI.

Method	Result(s) for best model	Regions	Factors	Author(s) and Year
Adaboost, Catboost, GB, KNN, Linear Regression, RF, SVM, XGBoost	For AQI: R^2^ = 0.9850, RMSE = 11.2696, MAE = 8.3845 (XGBoost)	Azamgarh, India	PM_2.5_, PM_10_, NO_2_, SO_2_, T, Humidity, WD, WS, UV Radiation	[22]
KNN, SVM, DT, RF, MP, GB, LR, SM	For AQI: R^2^ = 0.973, RMSE=7.568, MAE=4.596 (SM)	Beijing, China	PM_2.5_, PM_10_, NO_2_, SO_2_, CO, O_3_	[23]
LightGBM, RF, CatBoost, AdaBoost, XGBoost	For AQI: R^2^ = 0.9998, RMSE = 0.76, MAE = 0.60 (CatBoost)	Visakhapatnam, India	PM_2.5_, PM_10_, NO_x_, NH_3_, SO_x_, CO, O_3_, Benzene, Toluene, Xylene, and meteorological factors	[24]
SVR, RFR, CR	For AQI: RMSE = 0.2792, Accuracy = 79.8622% of New Delhi (CR), RMSE = 0.5674 Accuracy = 68.6860% of Bangalore (CR), RMSE = 0.0988, Accuracy = 93.7438% of Kolkata (RFR), RMSE = 0.0628, Accuracy = 97.6080% of Hyderabad (RFR)	New Delhi, Bangalore, Kolkata, Hyderabad (India)	PM_2.5_, PM_10_, NO, NO_2_, NO_x_, NH_3_, CO, SO_2_, O_3_, Benzene, Toluene	[25]
SARIMA, SVM (RBF Kernel), LSTM	For AQI: R^2^ = 0.9989, RMSE = 4.944 (SVM with RBF kernel)	Ahmedabad city of Gujarat, India	PM_2.5_, PM_10_, NO_2_, SO_2_, CO, O_3_, NH_3_, Pb	[26]
LR, KNN, SVR, LSTM, RF, XGBT, LightGBM, LSTM-SVR (Hybrid)	For AQI: R^2^ = 0.994, RMSE = 3.277, MAE = 1.754 (LSTM-SVR)	Six major Chinese urban agglomerations	PM_2.5_, PM_10_, NO_2_, SO_2_, CO, O_3_, T, Pressure, Dew Temperature, WS, P	[27]
DT, RF, XGBoost	For AQI: R^2^ = 0.9214, RMSE = 29.6953, MAE = 18.9839 (XGBoost)	India	Xylene, PM_10_, NH_3_, Toluene, Benzene, PM_2.5_, NO_x_, O_3_, SO_2_, NO_2_, NO, CO	[13]
LR, KNN, SVM, LSTM, GRU, Hybrid LSTM-GRU	For AQI: MAE = 36.11, RMSE = 52.03, R^2^ = 0.84 (Hybrid LSTM-GRU)	Delhi, India	PM_2.5_, NO_x_, O_3_, SO_2_, NO_2_, NO, CO, T, WS, RH, Solar Radiation	[28]
DT, LogR, RF	For AQI: Accuracy = 98.63% (DT)	Uttarakhand state, India	PM_10_, PM_2.5_, SO_2_, NO_2_	[29]
ANN, MLR, ILSTM, SVR	For AQI: R^2^ = 0.981 (MLR)	Tamil Nadu State, India	PM_2.5_, PM_10_, SO_2_, NO_x_, NH_3_, CO and O_3_	[30]
SVR and LSTM	For AQI: RMSE = 10.995, R^2^ = 0.570 (LSTM)	Chennai city, India	PM_2.5_, NO_2_, SO_2_, CO, Ozon	[31]
SVR-RBF and PCA SVR-RBF	For AQI: accuracy = 94.1% (SVR-RBF)	California	CO, NO_2_, SO_2_, Ozone, PM_2.5_, WS, T, RH	[32]
SVR and RFR	For AQI: R^2^ = 0.9766 RMSE = 7.666 (SVR)	Beijing and Italian	PM_2.5,_ PM_10_, O_3_, SO_2_, NO_2_	[33]

As shown in Table 2, ensemble learning methods such as XGBoost, and LightGBM frequently achieved the highest accuracy in AQI prediction across different regions. Support vector-based models also provided competitive results in several studies, particularly for PM-based predictions. In most cases, particulate matter (PM_2.5_ and PM_10_) emerged as the dominant factor influencing air quality, highlighting its critical role in AQI forecasting. These studies demonstrate the growing interest in AQI prediction using machine learning. However, most of them focus on short-term datasets, metropolitan regions, or limited sets of pollutants and meteorological variables. To the best of our knowledge, no long-term, region-specific study has been conducted in a geopolitically sensitive area like Iğdır, Türkiye. In addition, existing models rarely integrate both pollutant and meteorological data over extended periods. This gap further highlights the novelty of the present research.

Air pollution prediction varies across different regions and climatic conditions due to the complex, nonlinear interactions between atmospheric pollutants and meteorological parameters, posing a significant challenge on a global scale [34–36]. Although machine learning models are widely used for AQI prediction, comprehensive evaluations of the forecast performance of these models are often limited to short-term datasets or specific subsets of pollutants [37–39]. This study aims to (i) reveal the long-term status and temporal dynamics of air pollution in Iğdır, Türkiye, during 2016–2024 using the TNAQI together with pollutant and meteorological records and (ii) systematically compare the predictive performances of advanced machine learning models (XGBoost, LightGBM and SVM) for daily AQI prediction and quantitatively evaluate the impacts of air pollutants and meteorological factors. Unlike traditional studies that usually focus on short-term datasets or specific subsets of pollutants, this research presents a comprehensive evaluation by integrating multiple air pollutants (PM_10_, SO_2_, NO_2_, O_3_) with key meteorological variables to improve the forecast accuracy. The findings provide valuable insights into the adaptability of machine learning models under different environmental conditions, suggesting a scalable and generalizable AQI prediction framework that can contribute to developing data-driven air quality management policies globally.

The study’s objectives are based on these observations:

a) Calculate daily AQI using data from four air pollutants (PM_10_, SO_2_, NO_2_, O_3_) and five meteorological parameters (temperature, precipitation, relative humidity, wind direction, wind speed) from 2016 to 2024.
b) Apply and compare advanced machine learning models, including XGBoost, LightGBM, and SVM, to predict the AQI with high precision.
c) Evaluate the performance of the machine learning models using metrics such as R², RMSE and MAE and assess their predictive capabilities.
d) Train the models on 80% of the dataset and validate them using the remaining 20% to ensure reliability and accuracy in AQI prediction.
e) Perform a comparative analysis to identify the most suitable model for AQI prediction, highlighting XGBoost’s consistent performance across all metrics.
f) Analyze model results to determine influential parameters impacting AQI predictions and offer insights for efficient air quality management.

This study offers a novel contribution to the field of air quality modeling by focusing on Iğdır province, a border region of Türkiye adjacent to three countries—Iran, Armenia, and Nakhchivan (Azerbaijan). Despite its strategic geopolitical location and vulnerability to cross-border pollution transport, the region has remained underrepresented in empirical AQI forecasting studies. By integrating eight years of daily meteorological and pollutant data (2016–2024), this study systematically compares the predictive performance of three advanced machine learning models—XGBoost, LightGBM, and SVM. The findings demonstrate that XGBoost significantly outperforms the other models in terms of accuracy and generalizability. This comprehensive regional analysis not only fills a critical gap in the existing literature but also provides a scalable framework for AQI prediction in other transboundary or climatically similar regions.

## Materials and methods

### Study area and data description

**Fig 1:**
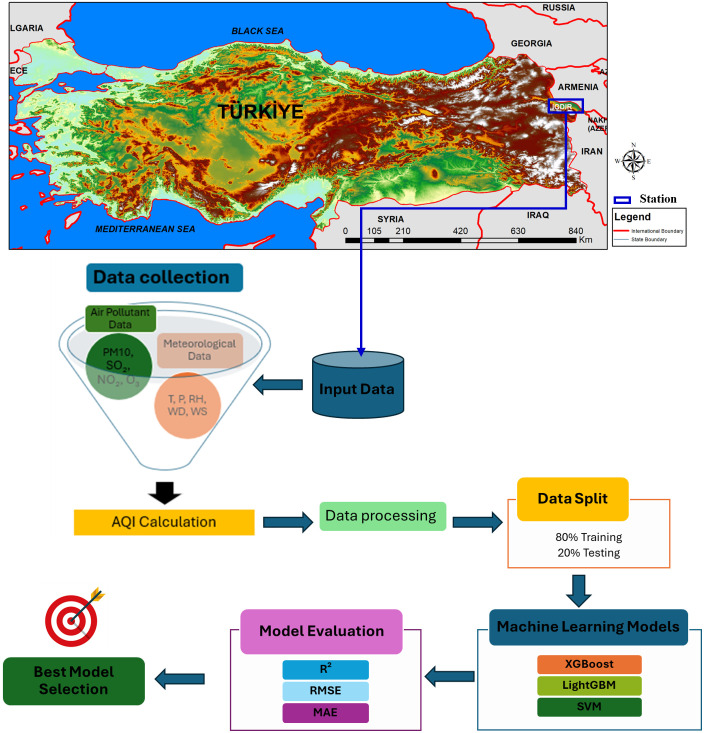
Location map of the study area and flowchart of the methodology. The study area is located in Iğdır Province, Türkiye. The base map is sourced from Natural Earth (public domain; http://www.naturalearthdata.com) and elevation data are from the USGS National Map Viewer (public domain; https://www.usgs.gov).

The current study examined the AQI of Iğdır City of Türkiye. Iğdır province is a settlement located east of Türkiye and borders three countries (Iran, Armenia, and Nakhchivan). The coordinates of Iğdır province are recorded as latitude: 39° 55′ 25.36″ N and longitude: 44° 02′ 42.00″ E. Its surface area is 3588 km^2^. Also, Iğdır, located at an altitude of 800–900 m, has a provincial population of approximately 210000. The geographical location of the study area is illustrated in Fig 1.

Air quality and meteorological data, including PM_10_, NO_2_, SO_2_, O_3_, wind speed, wind direction, relative humidity, temperature, and precipitation, were collected from air quality and meteorology stations operated by the Republic of Türkiye Ministry of Environment, Urbanization and Climate Change. The dataset comprises eight year daily air pollutants data (2016–2024) for Iğdır.

### Methodology for AQI calculation and indexing

In this study, AQI values were calculated using pollutant concentrations measured at the air monitoring station in Iğdır province. The TNAQI system, adapted to Türkiye’s national standards, was applied. According to this approach, the AQI is determined as the maximum sub-index among five pollutants (SO_2_, NO_2_, PM_10_, O_3_, CO). Reference intervals for pollutant concentrations and their corresponding AQI values are provided in Table 3 [16].

**Table 3:** Reference intervals used in AQI calculation.

AQI scale	Index value	SO_2_ [μg m^-3^] 1 h. Avg.	NO_2_ [μg m^-3^] 1 h. Avg.	CO [μg m^-3^] 8 h. Avg.	O_3_ [μg m^-3^] 8 h. Avg.	PM_10_ [μg m^-3^] 24 h. Avg.
Good	0–50	0-100	0-100	0-5500	0-120	0-50
Moderate	51–100	101-250	101-200	5501-10000	121-160	51-100
Unhealthy for sensitive groups	101–150	251-500	201-500	10001-16000	161-180	101-260
Unhealthy	151–200	501-850	501-1000	16001-24000	181-240	261-400
Very unhealthy	201–300	851-1100	1001-2000	24001-32000	241-700	401-520
Hazardous	301–500	>1101	>2001	>32001	>701	>521

The AQI calculation follows Equations (1) and (2). For this purpose, four pollutants (PM₁₀, SO₂, NO₂, O₃) measured in Iğdır were used:


$$AQI\;=\text{ max }(IAQI_{\text{1}},\;IAQI_{\text{2}},\;\ldots ,\;IAQI_{i})$$ (1)

$${IAQI}_{i}=I_{low}+\frac{C_{i}-C_{low}}{C_{high}-C_{low}}\left(I_{high}-I_{low}\right)$$ (2)

where i represents the air pollutant. IAQI_i_ is the air quality sub-index for pollutant i; C_i_ is the concentration of pollutant i; 𝐶_𝑙𝑜__𝑤_ and 𝐶_ℎ𝑖𝑔ℎ_ denote the minimum and maximum concentration values of the AQI category corresponding to the specific pollutant; 𝐼_𝑙𝑜__𝑤_ and 𝐼_ℎ𝑖𝑔ℎ_ denote the minimum and maximum AQI values for that category (see Table 3). The following calculations were used in the study: maximum one-hour average values (μg m^-3^) for NO_2_ and SO_2_, maximum eight-hour average values (μg m^-3^) for O_3_, and daily average values for PM_10_ (μg m^-3^). These daily AQI values, along with the daily average pollutant concentrations and meteorological parameters, were subsequently used as input for the machine learning models. The overall methodology is illustrated in Fig 1.

### Machine learning models

#### Light gradient boosting machine algorithm (LightGBM).

The LightGBM algorithm is a framework offering a gradient boosting method using tree-based learning methods [40]. It was developed by Microsoft researchers in 2017 [41]. This algorithm has a distributed structure and provides a faster training process and higher performance compared to other algorithms. LightGBM is based on a leaf-based growth strategy using one-sided gradient sampling, special feature grouping, and depth-limited histograms. This algorithm aims to achieve high prediction success by creating a strong learner from the combination of weak learners. In particular, LightGBM uses maximum depth-limited leaf-based growth and histogram-based methods to shorten the training time and reduce memory usage [42]. In addition, the histogram subtraction technique enables the use of a large number of histograms by dividing continuous explanatory variables, thus increasing statistical efficiency and accelerating convergence [43]. The LightGBM algorithm is a gradient boosting model built using tree-based classifiers [27,44,45]. Trees are constructed iteratively such that each step minimizes the loss function. However, traditional approaches often struggle with speed and capacity. To address these challenges, LightGBM efficiently handles large datasets and categorical features using techniques such as histogram-based splitting and leaf-wise tree growth [46].

#### eXtreme gradient boosting algorithm (XGBoost).

The XGBoost algorithm is a gradient boosting method proposed by Chen and Guestrin in 2016 [47]. The XGBoost aims to make more accurate predictions by adding a regularization term to the objective function to prevent overfitting [48]. The XGBoost algorithm constructs an ensemble containing a set of decision trees trained on different dataset partitions [49]. When splitting trees by depth or level, XGBoost determines the best branch splitting effect of each tree (decision) feature and the appropriate threshold for this feature. The XGBoost performs successive splits to make the tree structures more distinct [48]. Finally, the scores of the stable trees obtained during the training process are summed, and the final predicted value of the response variable is calculated [50].

#### Support vector machine algorithm (SVM).

The SVR algorithm is an important subgroup of support vector machine, one of the machine learning algorithms [51]. While the support vector machine algorithm used for classification operations is called support vector classification, the part that deals with modeling and prediction operations is called SVR [52,53]. Since SVR is a supervised learning method, the success of the predictions made with SVR varies depending on the training and test data sets [54]. The main goal of SVR in the linear SVR model is to define a function f(x) with the maximum deviation (ε) value from the training set and is as flat as possible. The training data lies within the limits between −ε and +ε [51]. However, many studies cannot be modeled within the framework of linear features. Therefore, in nonlinear SVR cases, the input data is transformed into a higher dimensional hilbert space so the regression line can be linear [54]. There are many nonlinear kernel functions, including the Gaussian radial basis function kernel. The kernel function used in this study is the Gaussian radial basis function kernel.

#### Model comparison criteria.

In this study, the performance of the models was assessed using three criteria: RMSE, MAE, and R².

**Root-mean-square error**. The RMSE is calculated as the square root of the Mean Squared Error (MSE), which represents the average squared difference between the observed actual output values and the model’s predicted values. RMSE provides a measure of how much the predictions deviate from the actual values, effectively serving as the standard deviation of these differences. It ranges from 0 to positive infinity, where a lower RMSE indicates predictions that are closer to the target values. In comparison, a higher RMSE reflects a more significant deviation and a wider distribution of values.


$$\begin{array}{c} RMSE=\sqrt{\frac{\text{1}}{n}\;\sum {\mathrm{\backslash nolimits}}_{i=\text{1}}^{n}{\hat{{(y}_{i}}-y_{i})}^{\text{2}}}\; \end{array}$$ (3)

**Mean Absolute Error**. The MAE quantifies the error in model predictions by calculating the mean of the absolute differences between the actual and predicted values. It is obtained by summing the absolute differences for all observations in the dataset and dividing this sum by the total number of observations. The MAE ranges from 0 to positive infinity, where smaller values closer to 0 indicate better performance, as they reflect that the predicted values are more closely aligned with the actual target values.


$$MAE=\frac{\sum_{i=\text{1}}^{n}|\hat{y_{i}}\;-\;y_{i}|}{n}$$ (4)

**R-Squared**. The R² represents how well the model predicts the target variable. This criterion ranges from 0 to 1. When R² equals 1, the predictions perfectly align with the data, indicating absolute accuracy, while lower R² values suggest weaker predictive performance.


$$R^{\text{2}}=\text{1}-\frac{\sum_{i=\text{1}}^{n}{\hat{{(y}_{i}}-y_{i})}^{\text{2}}}{\sum_{i=\text{1}}^{n}{{(\overset{―}{y}}_{i}-y_{i})}^{\text{2}}}\;$$ (5)

In the above equations, ŷ represents the predicted AQI variable, y denotes the actual AQI variable, $\overset{―}{y}$ symbolizes the average value of AQI, and i refers to the index of each data point in the dataset. The upper bounds of MAE and RMSE extend to positive infinity, which makes them less effective in fully reflecting a model’s overall performance across different datasets. In contrast, R², with its range confined between 0 and 1, provides a more reliable metric for comparing model performance on varying datasets. A model is considered effective when its RMSE and MAE values are low, and its R² value approaches 1. Furthermore, while all three metrics are suitable for evaluating a model’s performance on the same dataset, R² is particularly useful for comparing models across different datasets.

### Software information

The dataset was randomly split into 80% training and 20% testing. 10-fold cross-validation was used for all algorithms, and although different k values were tested, the most robust results were obtained with 10-fold validation. All statistical analyses were performed using R software [55]. The “psych” package was used for descriptive statistics, and the “corrplot” package was used to visualize the relationships between explanatory and response variables, and the “caret” package was used to separate the dataset into training and test sets [56–58]. For the implementation of LightGBM, XGBoost, and SVR algorithms, the “lightgbm”, “xgboost”, and “e1071” packages were preferred, respectively [59–61]. The feature importances of all models were visualized using the “ggplot2” package [62].

## Results

To estimate AQI, eight years daily air pollutant data (2016–2024) for Iğdır were used to compare different machine learning models. Table 4 presents descriptive statistics such as mean, standard deviation, minimum, and maximum values for all air quality criteria and meteorological features used in this study. For descriptive analysis and interpretation, daily measurements were aggregated into monthly averages, and the values shown in the table represent the mean monthly values across the eight-year dataset (2016–2024). This approach highlights seasonal variations and long-term trends. In the Table 4, *n* denotes the number of observations (sample sizes) used in the analysis within the scope of the statistical approach. For example, the average temperature in January was 1.67°C, with the lowest temperature being −11.8°C and the highest temperature being 7.7°C. In February, the average temperature increased slightly to 2.66°C. A significant rise in temperature was observed from March onwards, with the highest values recorded in July and August (27.28°C and 27.54°C). The temperature decreased from September onwards and was measured as 1.66°C in December (Table 4).

**Table 4:** Annual air pollutant and metrological factor dataset.

Parameters	Month	n	T	P	RH	WD	WS	PM_10_	SO_2_	NO_2_	O_3_	AQI
Mean	January	197	1.67	0.26	71.19	208.96	0.65	181.61	15.01	41.05	29	172.91
SD			4.58	1.1	11.21	149.83	0.45	109.95	11.3	13.97	11.95	74.23
Min			−11.8	0	41.2	1	0	14.12	2.41	7.69	4.7	18.59
Max			7.7	8.3	97.2	360	2.8	648.34	80.81	74.99	65.24	552.9
Mean	February	180	2.66	0.27	60.23	213.59	0.9	125.97	11.25	36.47	41.26	142.11
SD			5.1	1.91	13.89	147.04	0.63	72	6.62	15.85	15.22	56.95
Min			−10.9	0	29.7	1	0.2	17.11	2.98	6.69	12.63	22.55
Max			11.7	25.1	88	360	3.4	334.95	42.19	85.41	95	391.77
Mean	March	229	9.12	0.77	54.15	234.47	1.24	76.7	8.02	25.89	54.98	103.72
SD			3.62	2.8	13.93	136.51	0.7	51.67	3.91	11.67	16.77	63.26
Min			0.3	0	28.6	1	0.1	12.55	1.64	5.33	15.23	22.16
Max			19.4	27.5	92.2	360	3.4	335.37	28.71	58.3	115.04	325.47
Mean	April	206	14.72	0.85	50.09	204.06	1.19	65.86	5.67	21.71	58.73	95.63
SD			3.42	2.27	13.42	149.9	0.54	35.01	2.2	10.85	20.01	55.51
Min			5	0	26	1	0.2	10.23	0.65	6.64	16.65	25.33
Max			24.2	15.2	85.8	360	3.2	183.49	17.25	54.03	108.6	325.32
Mean	May	192	18.39	1.46	55.33	235.99	1.22	49.67	5.05	16.65	61.87	76.79
SD			3	2.84	13.12	141.29	0.49	25.89	2.26	6.39	22.46	52.17
Min			12.4	0	24.3	1	0.2	9.38	1.4	3.34	19.62	16.65
Max			26.7	19.3	84	360	3.6	142.35	12.14	36.55	115.96	304.69
Mean	June	161	24.13	0.77	47.33	244.76	1.25	64.35	5.13	15.82	73.32	99.47
SD			3.03	1.88	11.7	135.32	0.41	33.04	2.5	7.1	28.67	48.53
Min			17.3	0	20.8	1	0.4	12.4	1.6	4.77	16.29	27.01
Max			30.5	12.9	77.3	360	2.8	197.12	10.21	58.2	145.71	304.54
Mean	July	192	27.28	0.57	44.47	231.65	1.32	65.41	4.66	11.94	78.52	100.11
SD			2.55	1.69	8.79	147.7	0.52	35.81	2.06	3.67	28.38	49.72
Min			20.5	0	28.4	1	0.4	11.99	1.88	5.89	22.78	26.86
Max			33.1	14.8	78.2	360	3.1	189.75	11.45	23.36	137.95	325.14
Mean	August	211	27.54	0.19	42.89	214.02	1.06	85.85	4.96	15.35	70.09	128.83
SD			1.71	0.7	6.76	155.08	0.37	39.77	2.26	5.29	28.83	51.76
Min			21.1	0	28.5	1	0.2	25.07	1.76	5.76	15.43	34.87
Max			31.4	5.7	69.8	359	2.5	230.12	14.36	31.23	131.74	325.22
Mean	September	160	22.56	0.18	45.77	259.97	1.03	95	5.33	19.09	51.88	125.86
SD			3.2	0.98	8.67	129.88	0.57	52.45	3.31	7.01	20.53	54.63
Min			13.9	0	22.3	1	0.1	12.94	2.31	6.32	9.17	21.72
Max			29.8	10.3	81	360	2.8	234.37	15.8	41.11	92.53	325.26
Mean	October	137	14.46	0.66	63.35	229.42	0.59	94.87	5.57	27.78	30.57	118.27
SD			3.54	1.7	13.01	153.13	0.49	58.7	3.27	9.13	14.44	57.5
Min			5.1	0	36	1	0	12.22	2.1	6.73	7.43	16.46
Max			22.6	8.7	92.2	360	2.5	312.38	22.73	57.7	68.94	325.2
Mean	November	142	7.51	0.76	71.29	229.46	0.38	181.42	9.99	38.89	23.45	168.16
SD			3.14	3.05	11.72	158.82	0.36	89.11	6.29	11.56	13.35	52.11
Min			−1.7	0	38	1	0	14.06	2.4	7.62	4.38	22.05
Max			16.1	29.1	95.7	360	1.9	398.5	27.57	64.07	69.44	325.49
Mean	December	162	1.66	0.39	75.44	201.98	0.56	190.7	13.05	41.79	22.15	173.55
SD			4.36	1.58	12.47	158.47	0.57	105.89	7.39	16.83	13.03	59.69
Min	−13.2	0	36.1	1	0	31.13	3.95	5.95	3.27	31.13
Max	10.6	15.9	98.5	360	3.8	545.13	46.58	125.03	66.78	510.02

The precipitation parameter generally remained at low levels, with a maximum value of 29.1 mm measured in November. Relative humidity varied throughout the year, reaching its highest average of 75.44% in December. Wind speed generally remained low, with a maximum of 3.8 m s^-1^ measured in December. Wind direction generally varied between 200°-250° (Table 4).

The PM_10_ concentrations increased during the winter months and were determined to be 181.61 µg m^-3^ on average in January. The PM_10_ values decreased during the summer months but remained at high levels. SO_2_, NO_2_, and O_3_ concentrations also showed seasonal variation, with SO_2_ values increasing significantly during the winter months. The SO_2_ average was measured as 13.05 µg m^-3^ in December (Table 4).

When evaluated regarding AQI, the highest values were seen in January (172.91) and November (168.16), indicating that air quality was negatively affected in winter months. During the summer months, AQI remained at lower levels. All these results reveal that air quality in the region is strongly affected by seasonal changes and deteriorates in winter months. This situation can be attributed to increased fossil fuel use and meteorological conditions in winter months. The study makes a significant contribution to determining measures that can be taken to improve air quality in the region (Table 4).

**Fig 2:**
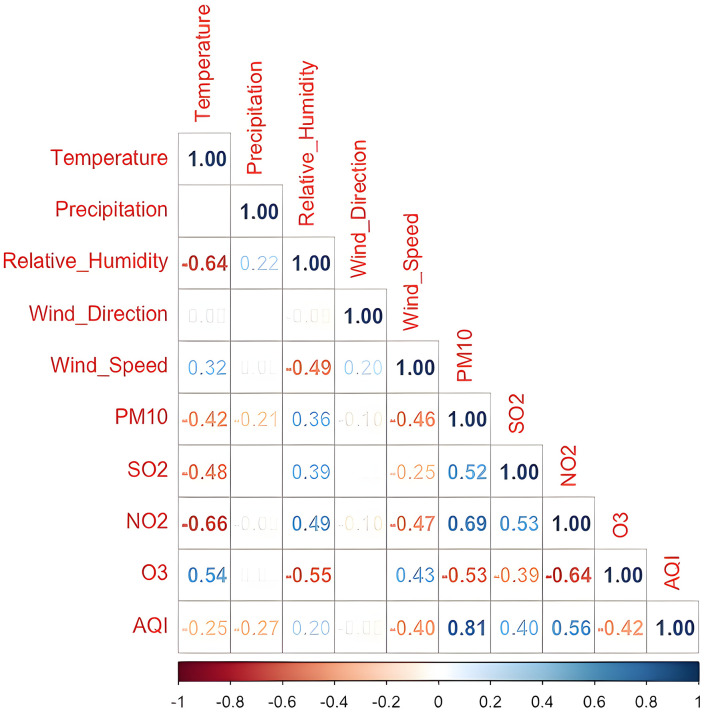
Correlation matrix for response and explanatory variables.

Fig 2 illustrates the correlation coefficients among environmental factors (temperature, precipitation, relative humidity, wind direction, wind speed, PM_10_, SO_2_, NO_2_, and O_3_) and their relationship with AQI. The coefficients range between −1 and +1, where the magnitude indicates the strength and the sign shows the direction of the relationship.

According to the correlation matrix, the highest correlation coefficient is between PM_10_ and AQI, with a coefficient of 0.81. In addition, a relatively high and positive correlation of 0.56 between NO_2_ and AQI indicates that increases in NO_2_ levels may be associated with increased AQI values. Similarly, the correlation coefficient 0.40 between SO_2_ and AQI is also noteworthy. However, the correlation of −0.25 between temperature and AQI suggests that temperature increases may be associated with decreased AQI values. Lower correlation values are observed between other variables (e.g., precipitation, relative humidity, wind direction) and AQI, indicating that the effect of these variables on AQI may be less pronounced.

**Fig 3:**
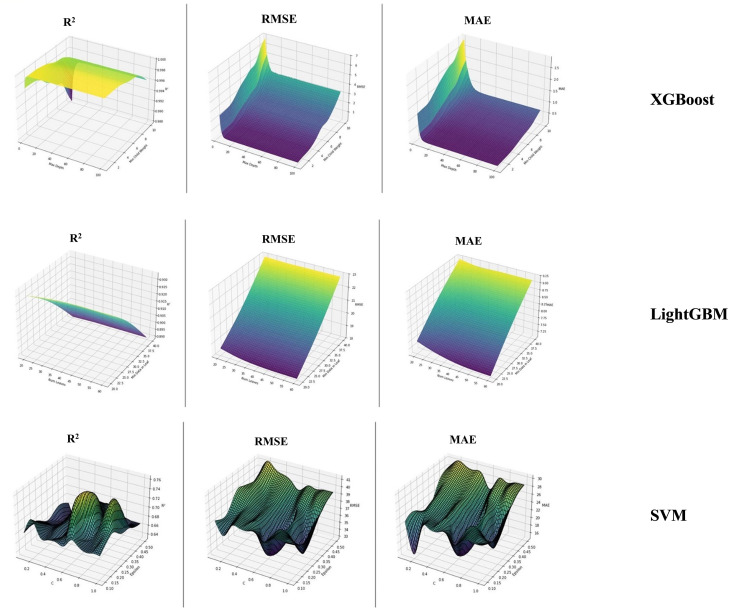
Optimized model results with 3D surface plot (Train set).

Hyperparameter optimization is a critical step in machine learning model development, as it directly influences predictive performance and generalization ability. Identifying the optimal parameter combinations ensures that the models are neither underfitted nor overfitted, thereby improving the reliability of AQI forecasts. Fig 3 presents the three-dimensional surface plots for the optimized model results on the train set, including the XGBoost (top), LightGBM (middle), and SVM (bottom) models. These plots illustrate the model performance under different hyperparameter combinations, where the x and y axes represent the hyperparameters, and the z-axis represents the corresponding performance metrics.

In the XGBoost section (top), the model performance shows relatively low variance across different hyperparameter settings, indicating stable results under specific parameter selections. The performance metric exhibits significant improvement and reaches peak values for certain hyperparameter combinations, highlighting the optimal conditions for the model (Fig 3). Additionally, error values are lower where the R² value is maximized, confirming the importance of model-specific hyperparameter tuning (Fig 3).

The LightGBM section (middle) includes three plots representing R², RMSE, and MAE metrics. The R² plot demonstrates how well the model explains variance in the dataset, with high values for specific parameter settings. The RMSE and MAE plots indicate the magnitude of prediction errors, where consistently low values across a wide range of hyperparameters suggest the model’s ability to make accurate and reliable predictions (Fig 3).

The SVM section (bottom) also displays R², RMSE, and MAE values, providing critical insights for optimizing the hyperparameters of the SVM model. The variations in these metrics highlight the model’s sensitivity to hyperparameter tuning, showing that proper selection of hyperparameters significantly impacts overall performance (Fig 3). The trends in these plots guide further optimization efforts to enhance the model’s predictive accuracy.

This comprehensive visualization allows for a comparative evaluation of the models, facilitating a deeper understanding of their respective performances under different parameter settings (Fig 3).

**Fig 4:**
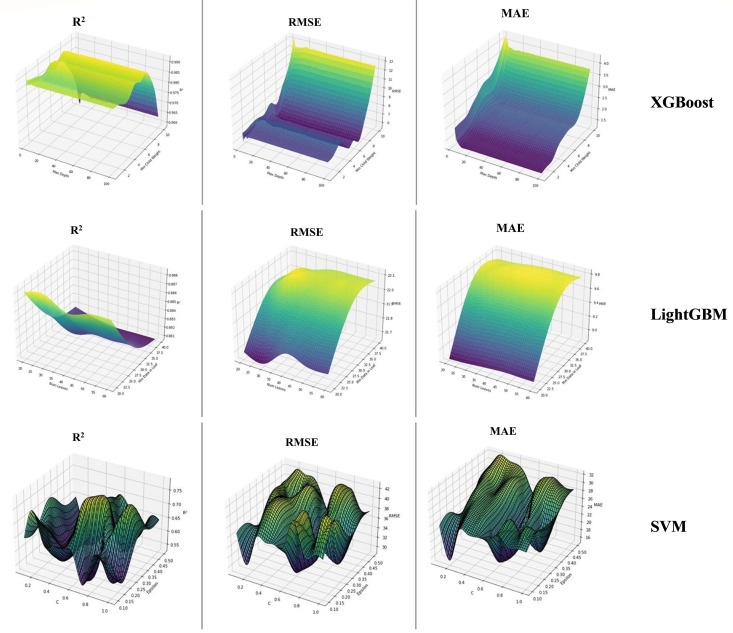
Optimized model results with 3D surface plot (Test set).

Fig 4 presents the three-dimensional surface plots for the optimized model results on the test set, showcasing the XGBoost (top), LightGBM (middle), and SVM (bottom) models. These visualizations demonstrate how model performance varies with different hyperparameter settings, where the x- and y-axes correspond to the selected hyperparameters and the z-axis reflects the associated performance metric, thereby highlighting the sensitivity of each model to parameter tuning.

In the XGBoost section (top), the performance metrics—R², RMSE, and MAE—demonstrate a pattern similar to the training set results. The consistency between the train and test sets indicates that the model maintains stable predictive capabilities across different datasets. Specifically, the plots reveal that high R² values correspond to lower RMSE and MAE, confirming that the selected hyperparameter combinations contribute to accurate model predictions (Fig 4).

The LightGBM section (middle) also provides insights into the model’s predictive success using R², RMSE, and MAE metrics. The R² plot highlights regions where the model effectively explains variance, particularly for certain hyperparameter settings. The RMSE and MAE plots indicate how prediction errors fluctuate under different conditions, with lower values signifying better performance (Fig 4). The model demonstrates robust generalization ability as it maintains low error rates across a broad range of hyperparameter settings.

The SVM section (bottom) displays performance variations based on different hyperparameter choices. The R² plot illustrates the model’s explanatory power, while RMSE and MAE plots highlight fluctuations in prediction errors. Notably, the wave-like structures in the plots indicate that the model is highly sensitive to specific hyperparameter selections. This sensitivity emphasizes the importance of fine-tuning to achieve optimal performance. Additionally, the results provide valuable insights into the model’s generalization capacity on test data, aiding in hyperparameter optimization (Fig 4).

By consolidating these models into a single Fig, Fig 3 facilitates a comparative evaluation, allowing for a clearer understanding of how each model performs on the test dataset under various hyperparameter settings.

Table 5 compares the goodness of fit criteria obtained with optimal hyperparameter values when LightGBM, XGBoost, and SVM algorithms are used in AQI estimation. LightGBM algorithm observed R^2^ values as 0.922 and 0.889 in training and test datasets, respectively, indicating that the model can explain the variance in the dataset to a large extent. RMSE and MAE values for the training set were measured as 18.661 and 5.666, respectively, while for the test set, these values were measured as 20.764 and 6.777, indicating that the model has slightly higher error rates in the test set.

**Table 5:** The goodness of fit criteria results in all algorithms for optimal hyperparameter values.

Hyperparameter values for each model							
LightGBM		XGBoost			SVM		
Learning rate	0.04	eta	0.1		C		1
Num leaves	30	Max depth	10		sigma		0.1
Min data in leaf	20	Min child weight	1		kernel		radial
**Goodness of fit criteria**							
	**LightGBM**		**XGBoost**			**SVM**	
**Criterion**	**Train**	**Test**	**Train**	**Test**		**Train**	**Test**
**R** ^**2**^	0.922	0.889	0.999	0.994		0.782	0.782
**RMSE**	18.661	20.764	0.234	4.84		28.824	31.136
**MAE**	5.666	6.777	0.158	0.972		12.233	13.546

The XGBoost algorithm performs better than other models, with R^2^ values indicating an almost perfect fit (0.999 in training, 0.994 in test). RMSE and MAE values are extremely low especially in the training set (RMSE 0.234, MAE 0.158), while these values are measured as 4.84 and 0.972 in the test set, suggesting that the model may have overfitted the training data. The SVM model showed an average fit with an R^2^ value of 0.782, which remained constant in both training and test sets; RMSE and MAE values were determined as 28.824 and 12.233 in the training set and 31.136 and 13.546 in the test set. These results show that the SVM model performs less than the other two models on this particular data set. As a result, the XGBoost algorithm stands out as the best-performing model in AQI estimation with high R^2^ values and low error metrics. The performances of LightGBM and SVM should be evaluated with more comprehensive analyses, especially in terms of generalization capabilities and error rates.

**Fig 5:**
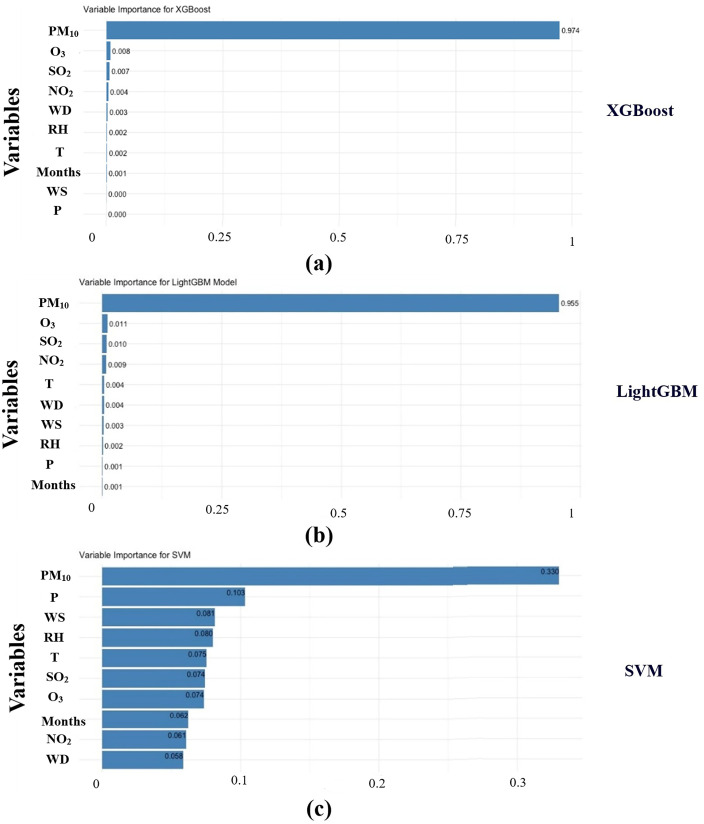
Variable importance for models. (a) XGBoost; (b) LightGBM; (c) SVM.

Fig 5 presents the variable importance levels for the three models—XGBoost (Fig 5a), LightGBM (Fig 5b), and SVM (Fig 5c)—and their respective impacts on AQI estimates. These plots highlight the significance of different variables in predicting AQI, offering insights into how each model prioritizes features.

In the XGBoost section (Fig 5a), PM_10_ emerges as the most influential variable with an importance score of 0.974, followed by O₃, SO₂, and NO₂ with scores of 0.008, 0.007, and 0.004, respectively. Other features, such as Wind Direction, Relative Humidity, and Temperature, hold lower importance levels, indicating their relatively minor impact on AQI predictions.

The LightGBM section (Fig 5b) presents a variable importance distribution similar to that of XGBoost. PM_10_ remains the most critical predictor with a score of 0.955, while O₃, SO₂, and NO₂ follow with importance values of 0.011, 0.010, and 0.009, respectively. Wind Direction, Wind Speed, and Temperature are identified as less significant features in the AQI estimation process.

The SVM section (Fig 5c) exhibits a different variable importance distribution compared to the other models. In this case, PM_10_ maintains the highest importance with a score of 0.330, but the ranking of secondary variables shifts. Precipitation, Wind Speed, and Relative Humidity gain prominence, with scores of 0.103, 0.081, and 0.080, respectively. Conversely, Temperature, SO₂, and O₃ hold lower importance levels, suggesting their diminished role in AQI predictions within this model. This finding is consistent with Choudhary et al. [63], who reported that various machine learning algorithms such as RF, SVM, Bagged MARS, and BRNN provided reliable predictions of particulate matter and gaseous pollutants, supporting the overall effectiveness of ML-based approaches in air quality studies.

A comparative analysis of these models indicates that XGBoost and LightGBM yield highly similar variable importance rankings, identifying the same key predictors for AQI estimation. This alignment reinforces the reliability of these models in accurately capturing influential environmental factors. Meanwhile, SVM assigns relatively lower importance to PM_10_ and emphasizes different meteorological variables, reflecting variations in how models process and interpret input features.

By consolidating these variable importance analyses into a single Fig, Fig 5 facilitates a clearer comparison across models, offering a comprehensive understanding of how each algorithm prioritizes different features in AQI estimation.

In the present study, the XGBoost model exhibits impressive goodness of fit with R^2^ values of 99.9% and 99.4% on the training and test sets, respectively, in various hyperparameter combinations. These high R^2^ values indicate that the model explains the variance in the data set extraordinarily well. The RMSE and MAE values of the XGBoost model are much lower than the other models, indicating that the estimates are closer to the actual values. Therefore, the model is more reliable. While the LightGBM and SVM models also exhibit consistent results, the superior performance of the XGBoost model suggests that it should be preferred more, especially in terms of the accuracy of the estimates and the general reliability of the model. In this context, it is concluded that the XGBoost model offers higher reliability and accuracy when making AQI estimates.

## Discussion

Monitoring and improving air quality is essential for both public authorities and individuals seeking to reduce environmental and health risks. Accurate AQI estimation supports these efforts, and machine learning algorithms provide powerful tools for this task. In this study, three algorithms—SVM, LightGBM, and XGBoost—were applied to AQI prediction using pollutant and meteorological data from Iğdır, Türkiye. All three models achieved satisfactory performance, but XGBoost consistently outperformed the others, yielding R² = 0.999, RMSE = 0.234, and MAE = 0.158. These results demonstrate that XGBoost is an effective and reliable model for AQI prediction, with reduced risk of overfitting compared to previous approaches.

These findings confirm that XGBoost has a superior ability to handle particularly complex datasets and understand the interactions between multidimensional features. Similar performance of XGBoost has also been reported in other studies [64,65]. For example, Van et al. [13] compared the performances of Decision Tree, Random Forest, and XGBoost algorithms. They stated that the XGBoost model gave the best results in accuracy (R² = 0.9993), error (RMSE = 2.5359), and MAE (1.2844) metrics on two different datasets.

Although the LightGBM model showed lower performance than XGBoost, it provided a good level of accuracy in AQI prediction. R² values for LightGBM were recorded as 0.922 in the training set and 0.889 in the test set. RMSE values were 18.661 and 20.764, and MAE values were 5.666 and 6.777, respectively. These findings show that LightGBM offers an important alternative for relatively less complex models. Ravindiran et al. [25], in particular, stated that CatBoost stood out in their similar studies, but LightGBM also produced reliable results in most cases.

The SVM model, on the other hand, exhibited lower performance compared to the other two models. The R² value was recorded as 0.782 in both training and test sets. The RMSE values were 28.824 and 31.136, and the MAE values were 12.233 and 13.546, respectively. These results indicate that SVM should be optimized for more complex datasets and multidimensional features. Liu et al. [33] emphasized that an SVR-based model was successful in some cases, but its generalizability may be limited.

The challenges associated with traditional air quality monitoring methods further emphasize the importance of using machine learning for AQI estimation. Traditional approaches often rely on fixed monitoring stations, which can provide limited spatial coverage and cannot effectively capture local pollution events [66,67]. In contrast, machine learning models play a pivotal role in integrating diverse datasets, including real-time sensor data and historical pollution records, to improve the accuracy of predictions. This comprehensive approach is particularly important in urban environments, where pollution sources can vary significantly in different areas [68,69]. Moreover, integrating meteorological parameters into predictive models is crucial because weather conditions such as temperature, humidity, and wind speed can significantly affect pollutant distribution and concentration levels [70,71]. The impact of meteorological parameters (temperature, precipitation, wind direction, wind speed, and humidity) on air quality is important in this study. The findings showed that meteorological factors are important inputs for AQI estimation, and air pollution varies depending on environmental conditions. Sigamani and Venkatesan [30] stated that meteorological factors play an important role in AQI estimation, affecting pollution concentrations by 60–74%.

This study’s findings align with various machine learning-based AQI prediction studies in literature. Ravindiran et al. [24] highlighted that the CatBoost model performed best with R² = 0.9998 and RMSE = 0.76; however, in this study, the performance of XGBoost is close to CatBoost. Similarly, Liu et al. [33] found that an SVR-based model was superior in AQI prediction (R² = 0.9766), but the study did not cover newer algorithms such as XGBoost.

The study’s findings also contribute to the ongoing discourse on overfitting in machine learning models. Overfitting occurs when a model learns from the noise in the training data rather than the underlying patterns, leading to poor generalization of unseen data. The ability of the XGBoost model to maintain consistent performance across a range of metric scores suggests that it effectively mitigates overfitting, a concern noted in previous research on machine learning applications in environmental science [72,73]. This feature is particularly valuable in the context of air quality prediction, where accurate prediction is essential for public health interventions and policymaking.

Moreover, the implications of improved AQI prediction extend beyond purely academic interest; there are real-world applications in public health and urban planning. Accurate AQI predictions can inform government responses to pollution events, enabling timely public health warnings and interventions. For example, during periods of high pollution, authorities can impose traffic restrictions or encourage public transportation to reduce exposure risks [74,75]. Additionally, individuals can use AQI predictions to make informed decisions about outdoor activities, reducing the health risks associated with poor air quality [76–77]. In this regard, Kumar et al. [78] further noted that conventional AQI measures may underestimate actual health risks, highlighting the importance of developing reliable forecasting systems that can better inform public protection strategies.

The study’s results are significant, particularly in the context of recent global events such as the COVID-19 pandemic. During the pandemic, several studies demonstrated that poor air quality aggravated respiratory conditions and increased vulnerability to severe outcomes from SARS-CoV-2 infection [79,80]. In this regard, the ability to accurately predict AQI is especially valuable, as it can support early interventions and public health strategies aimed at reducing exposure to harmful pollutants during health crises. This makes the study’s findings highly relevant for protecting public health, particularly in densely populated urban areas where pollution levels are often elevated.

Most of the studies summarized in Table 2 focus on short-term datasets, a limited number of pollutant variables, or exclusively metropolitan regions. Moreover, many of these studies do not incorporate the long-term combined evaluation of meteorological and pollutant parameters. In this context, our study contributes to literature by using a long-term dataset that integrates both pollutant and meteorological variables. Conducting such a long-term analysis in a geopolitically sensitive and climatically unique region like Iğdır further reinforces the originality and significance of our findings.

In conclusion, the application of machine learning algorithms, particularly XGBoost, to predict AQI in Iğdır, Türkiye, demonstrates the potential of these technologies to improve air quality management. The study’s findings not only highlight the effectiveness of machine learning in this area but also highlight the importance of integrating diverse datasets and addressing overfitting to increase model reliability. As urbanization continues to increase and air pollution remains a pressing global issue, developing robust predictive models will be important to create healthier environments and support public well-being.

## Conclusion

Air pollution levels in Iğdır have increased significantly, particularly during the winter months. The average AQI values in January and November were 172.9 and 168.2, respectively, which correspond mostly to the “Unhealthy” category according to the TNAQI classification. PM₁₀ was determined to be the primary pollutant in pollution.

Analysis and model comparison results indicate that the XGBoost model achieved significantly superior performance in AQI predictions compared to the LightGBM and SVM models. While the R^2^ values of the XGBoost model were exceptionally high in both the training (99.9%) and test (99.4%) sets, the error metrics, such as RMSE and MAE, were also notable with low values such as 0.234 and 0.158, respectively. These results show that the model explains the variance in the dataset perfectly well, and the predictions are highly accurate.

On the other hand, the LightGBM model also exhibited robust results, with R^2^ values measured as 92.2% and 88.9% in the training and test sets, respectively. This model also provided acceptable performance with low RMSE and MAE values. On the other hand, the SVM model exhibited lower reliability than these two models, with R^2^ values remaining at 78.2% in both sets and relatively high RMSE and MAE values, indicating that the model is not as effective as the others.

Considering these findings, the XGBoost model offers a more reliable and effective alternative for AQI estimations than other models. These results underline XGBoost’s high adaptability and accuracy capacity, especially in complex data structures and when evaluating the interaction of various environmental parameters. The proposed approach can serve as a valuable guide for future modeling studies and constitute a basis for applications on larger data sets.

## Abbreviations

RMSE: Root Mean Square Error
MLR: Multiple Linear Regression
MSE: Mean Squared Error
MAE: Mean Absolute Error
R^2^: Coefficient Of Determination
SD: Standard Deviation
T: Temperature
P: Precipitation
RH: Relative Humidity
WD: Wind Direction
WS: Wind Speed
RF: Random Forest
RFR: Random Forest Regression
CR: Catboost Regression
DT: Decision Tree
MP: Multilayer Perceptron
GB: Gradient Boosting
LR: LinearR, Lasso Regression
SM: Stacked Models
KNN: K-Nearest Neighbor
LightGBM: Light Gradient Boosting Machine
SVR: Support Vector Regression
LSTM: Long Short-Term Memory
LogR: Logistic Regression
MLR: Multiple Linear Regressive
ANN: Artificial Neural Networks
ILSTM: Improved Long Short-Term Memory Algorithm
BRNN: Bayesian Regularized Neural Networks
